# Exploring Association Between Serotonin and Neurogenesis Related Genes in Obsessive-Compulsive Disorder in Chinese Han People: Promising Association Between DMRT2, miR-30a-5p, and Early-Onset Patients

**DOI:** 10.3389/fpsyt.2022.857574

**Published:** 2022-05-13

**Authors:** Miaohan Deng, Yuan Wang, Shunying Yu, Qing Fan, Jianyin Qiu, Zhen Wang, Zeping Xiao

**Affiliations:** Shanghai Mental Health Center, Shanghai Jiao Tong University School of Medicine, Shanghai, China

**Keywords:** single locus polymorphism, obsessive-compulsive disorder, BDNF, DMRT, serotonin

## Abstract

Obsessive-compulsive disorder (OCD) is a deliberating disorder with complex genetic and environmental etiologies. Hypotheses about OCD mainly include dysregulated neurotransmitters, especially serotonin, and disturbed neurodevelopment. Single nucleotide polymorphism (SNP) association studies regarding OCD are often met with inconsistent results. However, stratification by age of onset may sometimes help to limit the heterogenicity of OCD patients. Therefore, we conducted a stratified SNP association study enrolling 636 patients and 612 healthy controls. Patients were stratified by age of onset as early-onset (EO-OCD) and late-onset (LO-OCD). Blood extracted from the patients was used to genotype 18 loci, including serotonin system genes, Slitrk1, Slitrk5, and DMRT2 and related miRNA genes. Logistic regression was used to compare allele and genotype frequencies of variants. A general linear model was used to evaluate the association between variants and trait anxiety. In our study, rs3824419 in DMRT2 was associated with EO-OCD, G allele was the risk allele. Rs2222722 in miR-30a-5p was associated with EO-OCD, with the C allele being the risk allele. Rs1000952 in HTR3D was found associated with trait anxiety in OCD patients. The significance disappeared after FDR correction. Our results supported neurodevelopment-related genes, DMRT2 and miR-30a-5p, to be related to EO-OCD. However, we cannot prove serotonin genes to be directly associated with EO-OCD. While an association between HTR3D and trait anxiety was discovered, comparisons based on biological or clinical traits may be helpful in future studies. As our detective powers were limited, more large-scale studies will be needed to confirm our conclusion.

## Introduction

Obsessive-compulsive disorder (OCD) is one of the most common mental disorders, with a prevalence of 2–3% worldwide (1), and 2.4% in China (2). Genetic and environmental factors are both involved in the etiology and pathogenesis with the heritability as high as 26–45% (3). It is characterized by recurrent intrusive thoughts (obsessions) and corresponding repetitive behavior (compulsions), which may be explained by the chaos of neurotransmitters (1).

Due to the early finding that OCD patients respond to selective serotonin reuptake inhibitors (SSRIs), most of the etiological studies in OCD focused on serotonin-related systems. Numerous studies were conducted on serotonin-related candidate genes, including serotonin receptors (HTR), serotonin transporter (SERT, or SLC6A4, or 5-HTT), tryptophan hydroxylase 2 (TPH2), and so on. Meta-analyses showed that serotonin 2a receptor (HTR2A) and serotonin transporter-linked polymorphic region (5-HTTLPR) got the most evidence in single nucleotide polymorphisms (SNPs) studies in OCD (4, 5). However, the results of genetic association studies exploring other serotonin-related genes were inconsistent.

After stratified analysis by age of onset, some clearer clues appeared. Early-onset OCD (EO-OCD), as a putative subtype of OCD, is increasingly regarded to have unique biological characteristics (6). Given its higher heritability and distinct comorbidity patterns, EO-OCD was more suggested to be a neurodevelopmental disorder caused by the perturbation in neurodevelopment processes than late-onset OCD (LO-OCD) (7). Neurodevelopment processes include axon guidance and dendrite development prenatally, as well as synaptic plasticity postnatally.

Brain-derived neurotrophic factor (BDNF) is one of the most studied biomarkers based on the neurodevelopmental hypothesis. It is a protein involved in the formation of the nervous system, playing a key role in cortisol development and synaptic plasticity (8). It may interact with the neurotransmitter systems (9), underlying the pathogenesis of a wide range of mental disorders. Altered levels of BDNF were observed in OCD. But results were different between studies in adults and children, while BDNF levels decreased in adult OCD patients and increased in children with OCD (10–13). These clues suggested that there are likely some complex links between BDNF and OCD. SNP rs6265 (or Val66Met), which was the most studied SNP in BDNF, is the only known functional SNP in the BDNF gene. Its relationship with OCD was uncertain. Meta-analyses of previous studies detected no or ethnicity-specific weak association between OCD and rs6265 (14, 15). Homogenous samples are needed for further certification.

Slitrks (SLIT and NTRK like family members), comprising a series of neurogenesis-related proteins including Slitrk1-6, are also promising candidate genes in OCD. All of them were highly expressed in the human brain, regulating neuronal outgrowth, neuronal survival, and synapse formation (16). Slitrk1 was discovered to be related to Tourette syndrome (16). Tourette syndrome is a neurodevelopmental disorder that may show some overlap with OCD in pathogenesis. Slitrk5 was also thought to be related to OCD. A study, which showed that slitrk5 knock-out mice manifested OCD-like phenotypes, suggested strongly that Slitrk5 may be involved in the onset of OCD (17).

DMRTs (doublesex and mab-3-related transcription factors), including DMRT1-8, were also potential candidate genes. They play a conserved role in sexual development and other developmental processes like neural development (18). According to current literature, DMRTs were essential genes for the central nervous system during early embryonic development, playing critical roles in brain patterning, corticogenesis, and other neurogenesis processes (19, 20). Previous studies suggested a putative gene in distal 9p associated with OCD (21, 22). DMRT2 is right located in the DMRT1-DMRT3-DMRT2 gene cluster in 9p24.3, the most susceptive location. In mice, it was detected to mostly express in adult testis and brain (23) but its expression condition and its polymorphisms in the OCD population have not yet been studied.

MiRNAs are non-coding RNAs regulating gene expression post-transcriptionally *via* mRNA degradation or transcription inhibition. MiRNAs are increasingly getting attention in mental disorders because they regulate up to 60% of protein-coding genes (24) and are highly expressed in the developing central nervous system (25). Since its function of orchestrating genetic spatiotemporal expression in the transitional processes during neurodevelopment, it provides a different perspective to elucidate the complex role of neurotransmitter chaos in mental disorders. Also, it was found to be related to several neurodevelopmental diseases such as Autism Spectrum Disorder (ASD) and schizophrenia for its continuous effect on the process of neurogenesis and synaptic plasticity (26, 27). A few studies revealed the value of circulating miRNAs supposed to be biomarkers of OCD (28, 29). Evidence has shown that miRNA-directed regulation in behavioral disorders can be affected by SNPs (30). Nevertheless, miRNA gene polymorphisms in OCD had not yet been reported.

In this study, we chose candidate genes, including serotonin-related genes (HTR1B, HTR3A/B/D/E, SERT, TPH2), BDNF, DMRT2, Slitrk1, and Slitrk5, in order to verify and explore the SNP association study in OCD. To elucidate the potential influence of MiRNA, we selected MiRNA which regulates these candidate genes. The selected MiRNA were hsa-miR-96 (HTR1B), hsa-mir-497 (HTR2A), hsa-mir-544a (HTR3D), hsa-mir-544b (HTR3D), hsa-mir-195 (BDNF), and hsa-mir-30a-5p (BDNF)^1^. To better demonstrate the research landscape of our chosen SNPs, literature about these SNPs in human sapiens is presented in Supplementary Table 1.

## Methods

### Subjects

Samples from 636 OCD patients and 612 healthy controls were collected from the biobank of Shanghai Mental Health Center (31). All the patients were diagnosed by a psychiatrist according to DSM-IV in the Chinese Han population. The Mini-International Neuropsychiatric Interview (M.I.N.I.) was used to check the diagnosis (32). Those OCD participants were included if they met the following criteria: (1) met the DSM-IV diagnostic threshold OCD; (2) had a Yale-Brown Obsessive-compulsive Scale (Y-BOCS) total score ≥16 (33); (3) were between 18 and 64 years old; (4) had at least an elementary school education; (5) were in sufficient health to complete the research. Exclusion criteria include: (1) being pregnant; (2) being previously or contemporarily diagnosed as other mental disorders (e.g., affective disorder, schizophrenia); (3) having a history of intellectual disability or other neurological disorder. (4) having a severe physical health condition. (5) tic disorder.

All the patients underwent clinical assessment before blood extraction employing (1) the Yale-Brown Obsessive Compulsive Scale (Y-BOCS) to evaluate the severity of obsessive-compulsive symptoms (33); (2) the State–Trait Anxiety Inventory (STAI) including subscale State Anxiety Inventory (SAI) and Trait Anxiety Inventory (TAI) to evaluate state anxiety and trait anxiety (34); (3) the 24-item Hamilton Depression Rating Scale (HAMD24) to rate the severity of depression (35); (4) the Hamilton Anxiety Rating Scale (HAMA) to rate the severity of anxiety (36).

The study was approved by the ethics committee of Shanghai Mental Health Center. Written informed consents were obtained from all participants before enrollment.

### SNP Selection, DNA Extraction, and Genotyping

Nine SNPs, namely rs1000952 in HTR3D, rs7627615 in HTR3E, rs13212041, and rs6296 in HTR1B, rs6265 in BDNF, rs1176744 in HTR3B, rs1062613 in HTR3A, rs4570625 in TPH2, and rs1042173 in SERT(SLC6A4) were selected as they were SNPs reported to be related to OCD with mixed results or related to other psychiatric disorders, putative as risk SNP in OCD.

Other SNPs were selected according to the following steps. We selected SNPs in Slitrk1, Slitrk5, DMRT2 that presented: (1) MAF ≥0.10; (2) located within 5 kb to the 5'UTR/3'UTR of the target gene; (3) *r*^2^ >0.8 in the HapMap database (https://www.ncbi.nlm.nih.gov/snp/) with Chinese Han origin in the Beijing population. As none of the SNPs were reported before, one or two SNP in each gene was selected and rs3824419, rs17641078, rs9531519, rs9582391, in total four SNPs were selected from the preliminary results.

Candidate SNPs of miRNAs, including has-mir-544b(HTR3D), hsa-mir-30a-5p(BDNF), has-miR-96(HTR1B), has-mir-544a(HTR3D), hsa-mir-497 (HTR2A)/hsa-mir-195 (BDNF), were filtered in National Center for Biotechnology Information (NCBI) variation database (http://www.ncbi.nlm.nih.gov/variation/) according to: (1) located within 5 kb to the 5'UTR of target gene; (2) 1,000 Genomes MAF ≥ 0.05. As none of SNPs were reported before, one SNP in each gene was selected and finally rs10934682 in hsa-mir-544b, rs2222722 in hsa-mir-30a-5p, rs4421293 in hsa-miR-96, rs10144193 in hsa-mir-544a, rs78312845 in hsa-mir-497/hsa-mir-195, totally five SNPs were selected from preliminary results.

Genomic DNA was extracted from leukocytes in venous blood using the QiaAmp® Isolation system (Qiagen, Basel, Switzerland) with the guidance of standard protocol. SNP genotyping was performed on the MassARRAY® SNP IPLEX platform (Agena Bioscience™, San Diego, CA, USA). Detailed information about primer design and polymerase chain reaction process is available upon request. Quality control was performed by excluding individual SNPs or samples with genotype call rates lower than 95%, as well as SNP assays with poor quality spectra or cluster plots. Ten percent of samples were randomly tested on the same platform and no inconsistency was found.

### Statistical Analysis

**Figure 1:**
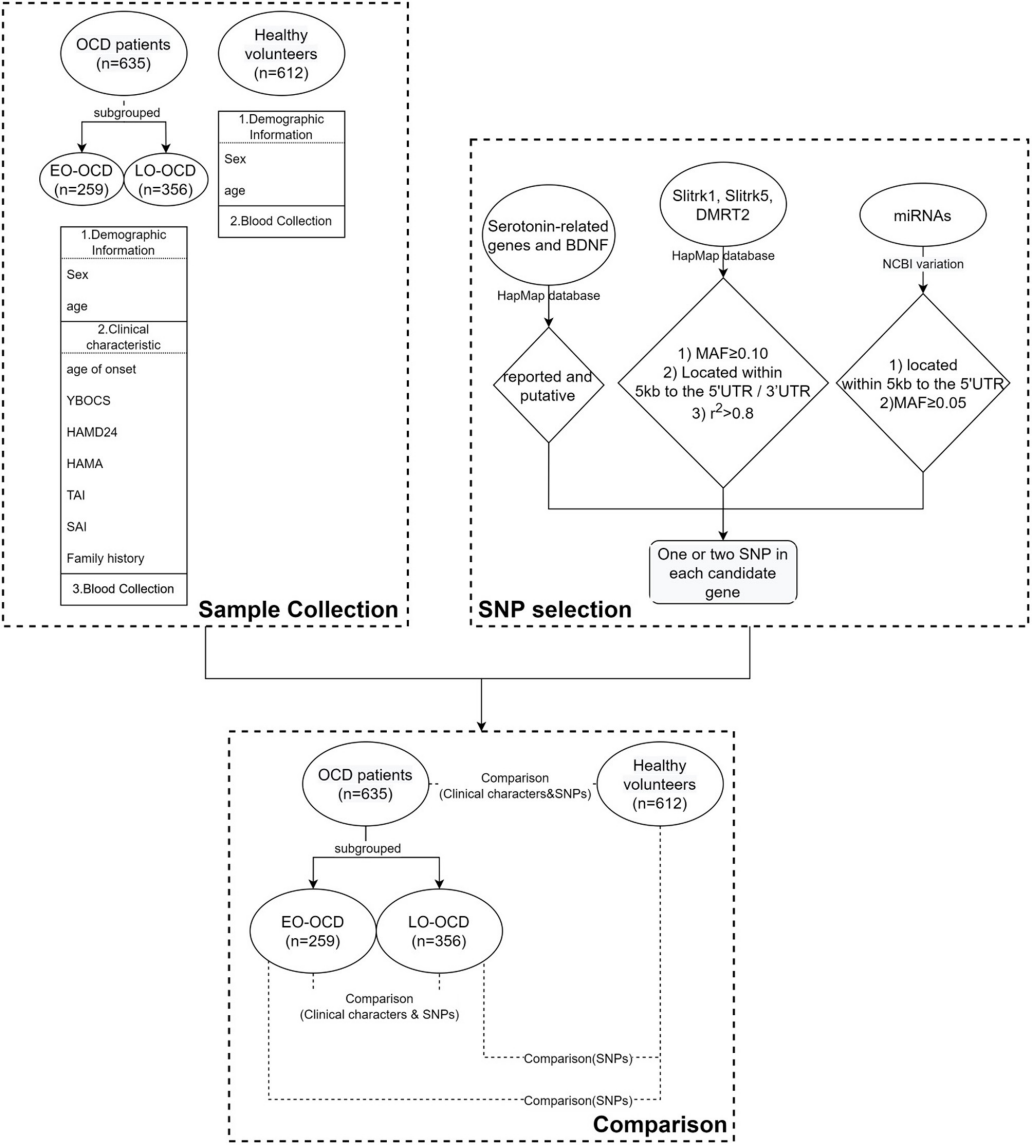
The flowchart of our work.

We used a two-tailed *t*-test, Mann–Whitney *U*-test, or Chi-square test to reveal the differences in demographic and clinical data. These results were presented as raw *p*-values. Hardy-Weinberg equilibrium (HWE) analyses were conducted using Haploview v4.2 (http://www.broad.mit.edu/mpg/haploview). Those which deviated from HWE were ruled out in the following analysis. In the following analysis, the OCD group was stratified as EO-OCD and LO-OCD by age of onset. According to previous studies and our clinical practice (37, 38), the cutoff between the EO and LO groups was set at 18 years old. General linear model analysis was used to evaluate SNP effects on TAI scores (adjust for sex and age of onset). All the single-locus analyses were analyzed by logistic regression (sex as a covariate) analysis employing an additive model for genotype analysis. The above process was conducted using SPSS Statistics v26.0 (IBM Corp., Chicago, IL, USA). We applied FDR correction for multiple testing. α was set as 0.05. Linkage disequilibrium and haplotype analysis were conducted using the SHEsis calculator (39, 40). Generalized multifactor dimensionality reduction (GMDR) analysis following 5,000 permutation tests was conducted to explore the gene^*^gene or gene^*^gene^*^sex interaction. The flowchart of our work was shown in Figure 1.

## Results

### Demographic Profile and SNP Characteristics

The demographic and clinical characteristics of participants were presented in Table 1. Sex (*p* < 0.001) and age (*p* < 0.001) were different between OCD and control. Sex (*p* < 0.001), age (*p* < 0.001), age of onset (*p* < 0.001), course of disease (*p* < 0.001), HAMD24 (*p* = 0.001), HAMA (*p* = 0.003), and TAI (*p* = 0.006) was different between EO-OCD and LO-OCD. The characteristics of SNPs are summarized in Table 2. Four SNPs including rs6296, rs4570625, rs10144193, and rs1042173 deviated from HWE (*p* < 0.05) and were excluded from the following analyses.

**Table 1:** Socio-demographic and clinical characteristics (Means ± SD).

**Variables**	**Overall OCD (*****n*** **= 635)**		**Control (*n* = 612)**	***P*-value**
	**Early-onset (*n* = 259)**	**Late-onset (*n* = 356)**		
Sex (male/female)	346/288		231/371	<0.001^***^
	164/95	169/186		<0.001^***^
Age (years)	30.23 ± 11.13		34.62 ± 12.06	<0.001^***^
	24.17 ± 7.05	34.33 ± 11.42		<0.001^***^
Age of onset (years)	22.96 ± 10.11		–	–
	15.29 ± 2.57	28.54 ± 9.88		<0.001^***^
Course of disease (month)	79.68 ± 81.67		–	–
	93.98 ± 84.88	65.46 ± 76.20		<0.001^***^
YBOCS	23.49 ± 7.14		–	–
	23.48 ± 7.67	23.09 ± 7.18		0.463
HAMD24	9.96 ± 5.56		–	–
	10.81 ± 5.69	9.18 ± 5.17		0.001^**^
HAMA	8.52 ± 5.19		–	–
	9.26 ± 5.07	7.87 ± 5.19		0.003^**^
TAI	47.63 ± 7.09		–	–
	48.27 ± 6.30	46.59 ± 6.89		0.006^**^
SAI	43.23 ± 7.93		–	–
	43.93 ± 7.70	42.65 ± 7.27		0.225

*Data are given as number or mean ± standard deviation*.

** *p < 0.01,*

*** *p < 0.001*.

*Bold values mean statistically significant values*.

**Table 2:** Characteristics of candidate genes and SNPs.

**Location**	**Gene symbol**	**Rs number**	**Wild genotype>variant allele**	**MAF**	**Group**	**HWE (*P*-value)**
3q21.2	hsa-mir-544b (HTR3D)	rs10934682	T>G	G = 0.167	OCD	0.3918
					Control	0.568
3q27.1	HTR3D	rs1000952	C>T	C = 0.091	OCD	0.9541
					Control	0.4219
3q27.1	HTR3E	rs7627615	G>A	G = 0.259	OCD	0.4984
					Control	0.9689
6q13	hsa-mir-30a-5p (BDNF)	rs2222722	C>T	T = 0.432	OCD	0.1181
					Control	0.6047
6q14.1	HTR1B	rs13212041	C>T	C = 0.244	OCD	0.0739
					Control	0.4172
		rs6296	G>C	G = 0.477	OCD	0.0012^**^
					Control	0.1119
7q32.2	hsa-miR-96(HTR1B)	rs4421293	G>A	A = 0.051	OCD	0.8951
					Control	1.0
9p24.3	DMRT2	rs3824419	G>C	G = 0.471	OCD	0.1031
					Control	0.8734
		rs17641078	G>C	C = 0.132	OCD	0.8364
					Control	0.5615
11p14.1	BDNF	rs6265	G>A	A = G = 0.5	OCD	0.0988
					Control	0.2477
11q23.2	HTR3B	rs1176744	T>G	G = 0.155	OCD	0.9151
					Control	0.1073
	HTR3A	rs1062613	T>C	T = 0.079	OCD	0.0727
					Control	1.0
12q21.1	TPH2	rs4570625	G>T	G = 0.489	OCD	0.0063^**^
					Control	0.0141^*^
13q31.1	Slitrk1	rs9531519	C>T	T = 0.331	OCD	0.2464
					Control	0.5895
13q31.2	Slitrk5	rs9582391	A>C	A = 0.243	OCD	0.6944
					Control	0.1543
14q32.31	hsa-mir-544a (HTR3D)	rs10144193	A>T	A = 0.271	OCD	0.1138
					Control	<0.001^***^
17p13.1	hsa-mir-497 (HTR2A)/hsa-mir-195 (BDNF)	rs78312845	G>A	G = 0.161	OCD	0.428
					Control	0.4423
17q11.2	SERT(SLC6A4)	rs1042173	T>G	T = 0.19	OCD	0.404
					Control	0.014^*^

* *p < 0.05,*

** *p < 0.01,*

*** *p < 0.001.*

*Bold values mean statistically significant values*.

### Overall OCD vs. Control

The genotype frequencies and outcomes of logistic regression are presented in Supplementary Table 2. There was no statistical difference observed in genotypic or allelic frequencies between OCD and the control group. No significant gene^*^gene or gene^*^gene^*^sex model was detected in GMDR analysis after the permutation test (data not shown).

### Early-Onset OCD vs. Control

Data from EO-OCD was compared with the healthy control. The results were demonstrated in Supplementary Table 2.

A significant difference was found both in the allelic and genotypic frequency of rs3824419 (genotype association *p* = 0.011; allele association *p* = 0.007). However, all the differences disappeared after FDR correction.

**Figure 2:**
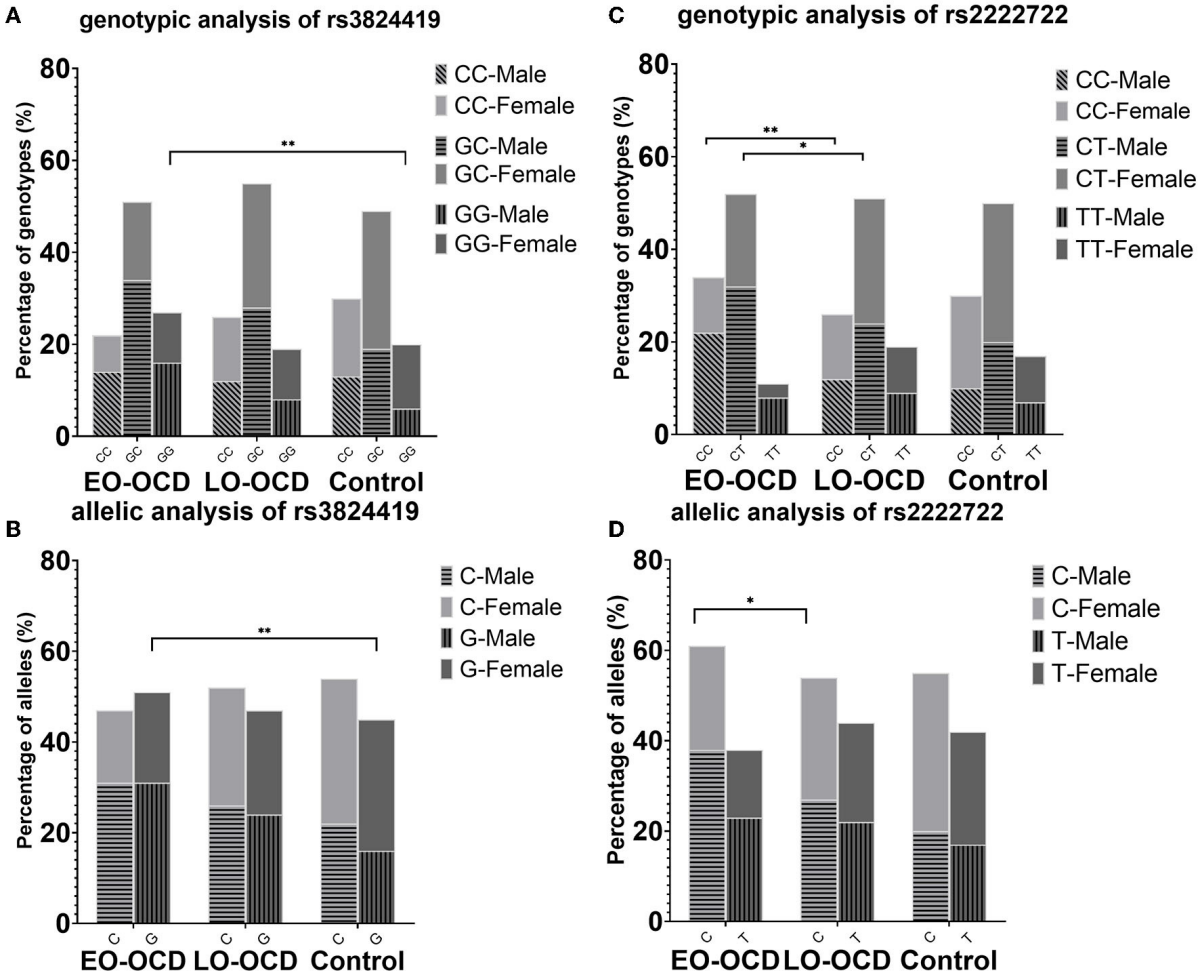
Genotype and allele association results of rs3824419 and rs2222722, adjusted and separately presented by sex. **(A)** Genotype association results of rs3824419. Between EO-OCD and control, GG homozygotes showed significantly higher risk. **(B)** Allele association results of rs3824419. Between EO-OCD and control, allelic distribution was significant difference and G allele showed higher risk. **(C)** Genotype association results of rs2222722. Between EO-OCD and LO-OCD, both CC homozygotes and CC heterozygotes showed significantly higher risk. **(D)** Allele association results of rs2222722. Between EO-OCD and LO-OCD, allelic distribution was significant difference and C allele showed higher risk.

With the significance of both allele and genotype analyses of rs3824419, we conducted further analysis on it, and the results are presented in Table 3. We observed a higher risk of G allele (OR = 1.375, 95%CI 1.089–1.736) and the increased risk of G carriers (CG, OR 1.341, 95%CI 0.925–1.943; GG, OR 1.935, 95% CI 1.256–2.982). Only the comparison in men manifested significance. These results are presented in Figure 2.

**Table 3:** Detailed results of sex-stratified analysis of rs3824419 and rs2222722.

**Rs number**	**Sex**	**Allele association**					**Genotype association**				
		**Allele**	**OR value**	***P*-value**	**95% CI**		**Genotype**	**OR value**	***P*-value**	**95% CI**	
					**Lower**	**Upper**				**Lower**	**Upper**
rs3824419 (EO vs. Control)	Sex-adjusted	G vs. C	1.375	0.007^**^	1.089	1.736	CC (contrast)		0.011^*^		
							CG	1.443	0.053	0.995	2.092
							GG	1.935	0.003^**^	1.256	2.982
	Male	G vs. C	1.394	0.036^*^	1.021	1.902	CC (contrast)		0.027^*^		
							CG	1.637	0.046^*^	1.009	2.654
							GG	2.194	0.009^**^	1.213	3.968
	Female	G vs. C	1.351	0.095	0.949	1.922	CC (contrast)		0.287		
							CG	1.199	0.535	0.676	2.127
							GG	1.635	0.126	0.871	3.070
rs2222722 (EO vs. LO)	Sex-adjusted	C vs. T	1.296	0.038^*^	1.014	1.657	TT (contrast)		0.013^*^		
							CT	2.161	0.003^**^	1.292	3.614
							CC	1.749	0.023^*^	1.081	2.830
	Male	C vs. T	1.341	0.074	0.972	1.851	TT (contrast)		0.079		
							CT	1.572	0.152	0.847	2.918
							CC	2.138	0.025^*^	1.100	4.154
	Female	C vs. T	1.236	0.273	0.846	1.806	TT (contrast)		0.140		
							CT	2.234	0.071	0.941	4.433
							CC	2.043	0.056	0.979	5.097

*EO, early-onset; LO, late-onset*.

* *p < 0.05;*

** *p < 0.01*.

*Bold values mean statistically significant values*.

In the following linkage disequilibrium analysis, rs3824419–rs17641078 showed different distribution between EO and control. The frequency of G-G is 0.523 in the EO group and 0.457 in the control (OR = 1.302, 95% CI 1.059–1.600) and that of C-G is 0.343 in the EO group and 0.412 in the control (OR = 0.744, 95%CI 0.601–0.922).

No significant gene^*^gene or gene^*^gene^*^sex model was detected (data not show).

### Late-Onset OCD vs. Control

Data from LO-OCD were compared with healthy control. The results were demonstrated in Supplementary Table 2.

No association of significance was observed in any analysis between LO and the control group (Supplementary Table 2). No significant gene^*^gene or gene^*^gene^*^sex model was detected (data not show).

### Early-Onset OCD vs. Late-Onset OCD

Single locus analysis revealed rs2222722 in miR-30a-5p to distribute differently between EO-OCD and LO-OCD (genotype association *p* = 0.013, allele association *p* = 0.038), while rs9582391 in Slitrk1 have only marginal allele and genotype association. All the significance disappeared after FDR correction (Supplementary Table 2).

We conducted further analysis on rs2222722 for the co-exist of genotype and allele association. We detected a higher risk of C allele in allele association (OR = 1.296, 95%CI 1.014–1.657) and an increased risk of C carriers (CT, OR 1.749, 95%CI 1.081–2.830; CC, OR 2.161, 95% CI 1.292–3.614). Both comparisons in men and women were of no significance. However, both comparisons showed a rather large OR value (Table 3). These results are presented in Figure 2.

No significant gene^*^gene or gene^*^gene^*^sex model was detected (data not shown).

### Relationship Between SNP and TAI Scores

**Figure 3:**
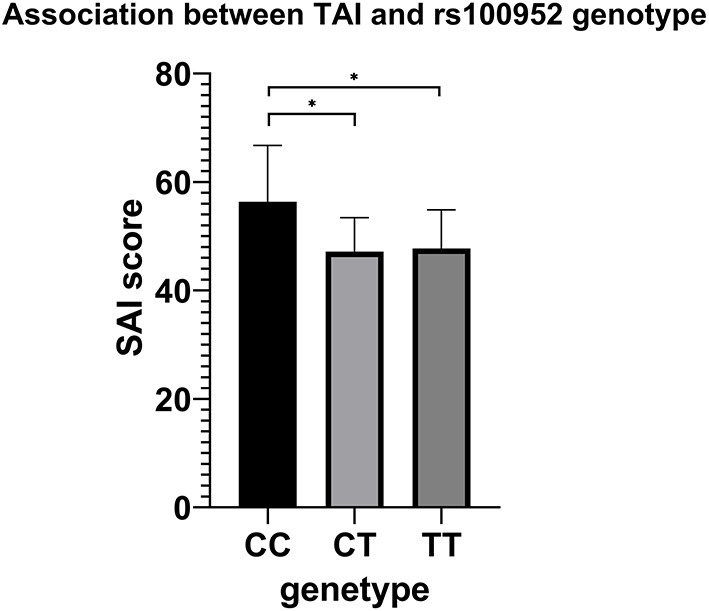
Association results between TAI and rs100952 genotype, adjusted by sex and age of onset. The general linear model revealed a significant difference (*F* = 3.744, *p* = 0.024). *p < 0.05 before FDR correction.

Comparison of TAI scores among genotype groups after adjusting for sex and age of onset were presented in Supplementary Table 2. Only rs1000952 in HTR3D demonstrated significance (*p* = 0.024), whereas the significance disappeared after FDR correction. The results were shown in Figure 3. In *post-hoc* analysis, patients carrying CC genotype presented higher TAI sore (60.68 ± 5.033) than CT (47.177 ± 0.695, Bonferroni's *P* = 0.03) or TT (47.740 ± 0.332, Bonferroni's *P* =0.018) while CT and TT were not different from each other (Bonferroni's *P* > 1). As OCD patients with CC genotype were extremely rare (*n* = 3), we did not make stratified analyses based on sex.

## Discussion

### Neurogenesis Related Genes Were Related to EO-OCD

Our results revealed rs2222722 in miR-30a-5p to be related to EO-OCD for the first time. Though the significance disappeared after FDR correction. In contrast, none of the positive results were observed between the overall OCD group or LO-OCD group and the control group. Our results corroborated the genetic heterogenicity of OCD and emphasized the importance of stratification by age of onset. Results from women and men showed similar trends when stratified by sex and the association was more significant in men. The impact of our relatively insufficient sample size in women should be considered. The reason why there was relatively fewer women than men in the EO-OCD group may be that EO-OCD presented a higher prevalence in men for unknown reasons (7). In the future, a larger sample size will be needed in female EO-OCD patients.

Our study found the DMRT2 haplotype (rs3824419–rs17641078) in strong linkage disequilibrium and its distribution was significantly different between EO-OCD and the control group. Both rs3824419 and rs17641078 are missense variants of the DMRT2 gene. The association analysis showed rs3824419 can well represent rs3824419-rs17641078 haplotype and it may be an important variant in DMRT2. To date, DMRTs are most famous for sex determination, and brain-related studies targeting DMRTs mainly focused on animal experiments. Among DMRTs, DMRT3 and DMRT5 were found to be highly expressed in the central nervous system, encoding nuclear proteins and playing an important role in neurogenesis (41). Evidence of DMRT2 was limited in the early linkage studies and 9p deletion-related studies. As was observed, catastrophic deletion of DMRTs may lead to severe functional disorders like intellectual disability in 9p deletion patients, or lead to abnormality in the macro-structure of the brain in knock-out mice (19, 20). SNPs with minor effects may also affect the brain at a smaller scale prenatally. There were several studies reporting that ASD was found in 9p deletion patients (42, 43). In fact, ASD was a well-known neurodevelopmental disorder sharing a genetic basis with OCD (44). Concerning the previous studies reporting a putative gene in distal 9p associated with OCD (21, 22), we deduced that DMRTs may play a role in the OCD pathogenesis. However, the exact effect of genetic factors and how they interact with prenatal environment factors remains to be explored. It is becoming clearer that DMRTs contribute to multiple aspects of CNS development, the mechanisms and pathologic relationship with human disease are still poorly understood. What exactly is the role of DMRT2 and other DMRT genes, especially DMRT1/3/5, in the onset of OCD, remained unknown. Our results suggested this gene family to be promising etiological genes of OCD, awaiting confirmation and mechanism research in the future.

We did not observe the significance of rs6265, the only known functional SNP in BDNF. This result was in accordance with our previous study (45). Though rs6265 may influence BDNF level with certain mechanisms, previous meta-analyses detected no or only ethnicity-specific weak association (14, 15). The locus rs2222722 came out instead. It is located in the miR-30a-5p gene in 6q13, downgrading the expression level of BDNF. MiR-30a-5p was previously found to present distinct developmental and lamina-specific expression in the human prefrontal cortex (46). Researchers found that miR-30a-5p in the prefrontal cortex was related to alcohol consumption (47) and miR-30a-5p in the striatum was related to Huntington's disease (48). EO-OCD occurs in a period when the brain is in intense development including neurogenesis, increased synaptic formation, and increased dendritic pruning. The dynamical fluctuation of miRNAs in different regions throughout their lifetime makes them switches or fine tuners in brain remodeling and neural plasticity before the age of 18 (49). Concerning neuroimaging studies that suggest distinct patterns of subcortical abnormalities in heterogeneous OCD patients (50), there may be different expressions of BDNF regulated by miRNA in different regions. Exploration of miRNAs has made great progress in other neurodevelopmental disorders like schizophrenia, Tourette Syndrome, and ASD (51, 52), but they were poorly studied in OCD. In our study, though we detected the risk allele, the pathway from SNP to miR-30a-5p, to region-specific expression of BDNF, and finally to the onset of OCD, remains to be clarified. In the context of the negative results of functional variant rs6265, our results called for attention to the potential role of miRNA and post-transcriptionally regulation of BDNF.

In our study, rs9582391 in Slitrk5 manifested marginal significance before FDR correction, but rs9531519 in Slitrk1 did not. While evidence in animal models was implicating the promising outlook of Slitrks (16, 53, 54), studies in human were disproportionately inadequate. A newly-published study distinguished Slitrk5 as the most significant single-gene result *via* exome sequencing of 1313 OCD participants (55), shedding light on the promising outlook of Slitrks. As we tested only two loci in this family, important SNPs may be missed. We were yet unable to determine the potential role of Slitrks in the pathogenesis of OCD in our study. This is the first time these two loci were reported in OCD. More explorations are needed, perhaps toward other SNPs, copy number variants, or rare variants.

### Serotonin System Variants Were Related to Trait Anxiety in OCD but Not Directly Related to EO-OCD

None of the serotonin-related SNPs was directly associated with OCD in our study, which may be due to the genetic complexity and phenotypic heterogeneity. On the one hand, though our analysis covered a wide range of common genes in the serotonin system, detected SNPs were limited in each gene. Important SNPs may be missed. On the other hand, a single gene may not play a vital role in OCD. For example, polymorphism of HTR1B was reported to be associated with a concentration of different neuro-metabolites in the anterior cingulate cortex in pediatric OCD patients (56), which indicates its relevance to the underlying pathology of OCD, but meta-analysis reported only a trend (4). While our analysis did not find any direct association between HTR1B and OCD, we conducted an analysis between related miRNA and OCD. Rs13212041 was a functional variant affecting miRNA-mediated HTR1B regulation in aggression-related behavior (30) and was associated with some neuropsychiatry characteristics (57) but its role in OCD has not yet been reported. Our result did not support its direct effect, either. There may be other important SNPs in the HTR1B system acting in other ways to affect the onset of OCD.

HTR3 genes were studied in very limited OCD studies. Rs1176144 in HTR3B was reported to be relevant to EO-OCD (58), rs7627615 in HTR3E, and rs1000952 in HTR3D were both reported to be associated with the washing phenotype of OCD (59), but we did not observe any significant direct association between them and OCD. Besides, our result on rs1062613 in HTR3A, a functional variant widely concerned to be associated with alcohol addiction, was in accordance with previous studies reporting a negative result on OCD (58, 60).

Trait anxiety is a personality trait presenting the predisposition of an individual to anxiety-related feelings or behaviors. It was found to correlate with reduced thickness in the medial orbitofrontal cortex in healthy volunteers (61). In OCD patients, trait anxiety may play a mediated role between OCD-related stress and functional disability (62). Though we did not find serotonin-related genes to be directly associated with OCD, we found rs1000952 in HTR3D related to trait anxiety and this SNP may exert influence on OCD through trait anxiety.

### Different Result Distribution Between Genes Related to Two Theory Systems

According to existing studies, comparisons based on specific bio-medical traits may be beneficial to limit the heterogeneity. A large-sample genetic association study made in a population-based, pediatric sample found 5-HTTLPR and HTR2A to be associated with rumination, one of the obsessive-compulsive trait symptoms (63), which also highlighted the importance of addressing symptom dimensions in OCD study. There were 14 subtypes of serotonin receptors identified, but it remained unclear whether and how each receptor affected OCD. Our analysis suggested an optional path to study the underlying pathology of serotonin receptors of OCD.

All the loci in our study were scarcely studied or never reported before in mental disorders. Though none of the results reached the FDR threshold, their different distribution in serotonin-related genes and neurodevelopment-related genes manifested an interesting trend. Besides the methodological reason, the underlying distinct neurobiochemical profiles are worthy of concern. Though serotonin disturbance is the most studied etiological theory in OCD, serotonin-related genes may cooperate with other neurotransmitter-related genes. Clinically, EO-OCD patients were thought to be less responsive to serotonin reuptake inhibitor therapy, requiring an augmentation treatment with antipsychotics (6). Neuroimaging data about serotonin transporter availability also suggested less serotonergic pathology in EO-OCD than LO-OCD (64), indicating more exploration from other perspectives. We were unable to decide the role of our chosen serotonin-related genes in OCD according to available data. However, neurogenesis-related genes, including BDNF-related genes, DMRTs, and Slitrks, may act in a more systematic way affecting considerable neurons and synapsis in neurotransmitter systems. Therefore, they may play a more vital role in the onset of EO-OCD with effects easier to detect.

### Limitations

Firstly, it was hard to avoid recall bias with regards to the age of onset under the retrospective background of our study. Secondly, as an exploratory analysis, this study covered a series of candidate genes but SNPs in each gene were limited, which may lead to the miss of important SNPs or haplotypes. Thirdly, due to the unavailable data, we did not make further analysis employing serum levels of biomarkers to draw the links between SNPs and neurochemicals. Fourthly, our power was limited in that the significance disappeared after FDR correction, though the study was conducted with a rather large sample size of 1,248 participants. As a result, confirmation with larger sample sizes was needed.

## Conclusion

To sum up, we found rs3824419 in DMRT2 and rs2222722 in miR-30a-5p to be related to EO-OCD. Our results suggested the merits of neurodevelopment-related genes in EO-OCD, supporting the neurodevelopmental hypothesis of EO-OCD and suggesting a distinct treatment strategy for it. In contrast, we did not find evidence of serotonin-related SNPs directly associated with OCD. Correlation between HTR3D and trait anxiety was observed, suggesting comparisons based on specific bio-medical traits in the OCD study. Finally, as our detection power was limited, more large-scale studies are needed to confirm our conclusion. To sum up, we found DMRT2, miR-30a-5p to be related to EO-OCD. Our results suggested the merits of neurodevelopment-related genes in EO-OCD, supporting the neurodevelopmental hypothesis of EO-OCD and suggesting a distinct treatment strategy for it.

## Data Availability Statement

The original contributions presented in the study are included in the article/Supplementary Materials, further inquiries can be directed to the corresponding author/s.

## Ethics Statement

The studies involving human participants were reviewed and approved by the Ethics Committee of Shanghai Mental Health Center. The patients/participants provided their written informed consent to participate in this study.

## Author Contributions

MD is responsible for original draft, visualization, and data curation. YW is responsible for the review, editing, funding acquisition, and data curation. SY is responsible for methodology. JQ and QF are responsible for resources collecting. ZW is responsible for project administration and funding acquisition. ZX is responsible for conceptualization, supervision, and funding acquisition. All authors contributed to the article and approved the submitted version.

## Funding

This work was supported by the National Natural Science Foundation of China (81971261), Hospital Project of Shanghai Mental Health Center (2019-YJ15), and the National Natural Science Foundation of China (82071518) in the sample collection and genotyping.

## Conflict of Interest

The authors declare that the research was conducted in the absence of any commercial or financial relationships that could be construed as a potential conflict of interest.

## Publisher's Note

All claims expressed in this article are solely those of the authors and do not necessarily represent those of their affiliated organizations, or those of the publisher, the editors and the reviewers. Any product that may be evaluated in this article, or claim that may be made by its manufacturer, is not guaranteed or endorsed by the publisher.

## Footnotes

^1^The gene name in the following bracket is the corresponding gene thought to be regulated by this miRNA.
