# Supplementary material for: Exploring Association Between Serotonin and Neurogenesis Related Genes in Obsessive-Compulsive Disorder in Chinese Han People: Promising Association Between DMRT2, miR-30a-5p, and Early-Onset Patients
Source: Front Psychiatry. 2022 May 13;13:857574. doi: 10.3389/fpsyt.2022.857574 (PMC9137639; doi:10.3389/fpsyt.2022.857574)
Supplement: Supplementary file 2 [file Table_2.docx]

Supplementary Material

# Supplementary Table 2 The results of single-site genotype and allele association analysis and the genotype association analysis of TAI scores

| Rs number | Group | Genotype count | Genotype association | | | | Allele association | | | | TAI association | | | |
| --- | --- | --- | --- | --- | --- | --- | --- | --- | --- | --- | --- | --- | --- | --- |
|  |  |  | Wald $\text{χ}^{2}$ | df | P value | FDR | Wald $\text{χ}^{2}$ | df | P value | FDR | F | df | P value | FDR |
| rs10934682 | Overall OCD | G G=22, G T=173, T T=440 | 1.544 | 3 | 0.462 | 1.000 | 0.360 | 2 | 0.549 | 1.000 |  |  |  |  |
|  | Early-onset | G G=8, G T=74, T T=176 | 0.528 | 3 | 0.768 | 1.000 | 0.000 | 2 | 0.996 | 0.996 |  |  |  |  |
|  | Late-onset | G G=12, G T=93, T T=251 | 1.386 | 3 | 0.500 | 0.938 | 0.000 | 2 | 0.995 | 0.995 |  |  |  |  |
|  | Control (Contrast) | G G=13, G T=173, T T=423 |  |  |  |  |  |  |  |  |  |  |  |  |
|  | EO versus LO |  | 0.537 | 3 | 0.765 | 1.000 | 0.050 | 2 | 0.822 | 0.948 | 0.318 | 4 | 0.727 | 1.000 |
| rs1000952 | Overall OCD | T T=528, C T=102, C C=4 | 0.386 | 3 | 0.825 | 0.990 | 0.289 | 2 | 0.591 | 1.000 |  |  |  |  |
|  | Early-onset | T T=216, C T=41, C C=1 | 0.506 | 3 | 0.777 | 0.971 | 0.046 | 2 | 0.831 | 1.000 |  |  |  |  |
|  | Late-onset | T T=296, C T=57, C C=2 | 0.416 | 3 | 0.812 | 1.000 | 0.639 | 2 | 0.424 | 1.000 |  |  |  |  |
|  | Control (Contrast) | T T=498, C T=107, C C=3 |  |  |  |  |  |  |  |  |  |  |  |  |
|  | EO versus LO |  | 0.156 | 3 | 0.925 | 1.000 | 0.069 | 2 | 0.793 | 0.991 | 3.744 | 4 | **0.024*** | 0.360 |
| rs7627615 | Overall OCD | A A=350, G A=245, G G=36 | 0.699 | 3 | 0.705 | 1.000 | 0.414 | 2 | 0.520 | 1.000 |  |  |  |  |
|  | Early-onset | A A=141, G A=102, G G=14 | 0.472 | 3 | 0.790 | 0.912 | 0.077 | 2 | 0.782 | 1.000 |  |  |  |  |
|  | Late-onset | A A=195, G A=138, G G=21 | 0.415 | 3 | 0.813 | 1.000 | 0.288 | 2 | 0.592 | 1.000 |  |  |  |  |
|  | Control (Contrast) | A A=326, G A=240, G G=43 |  |  |  |  |  |  |  |  |  |  |  |  |
|  | EO versus LO |  | 0.006 | 3 | 0.997 | 1.000 | 0.037 | 2 | 0.847 | 0.908 | 2.935 | 4 | 0.054 | 0.405 |
| rs2222722 | Overall OCD | C C=193, C T=335, T T=107 | 0.454 | 3 | 0.797 | 1.000 | 0.053 | 2 | 0.817 | 1.000 |  |  |  |  |
|  | Early-onset | C C=91, C T=136, T T=31 | 5.598 | 3 | 0.061 | 0.458 | 3.315 | 2 | 0.069 | 0.518 |  |  |  |  |
|  | Late-onset | C C=99, C T=184, T T=73 | 1.265 | 3 | 0.531 | 0.885 | 0.329 | 2 | 0.566 | 1.000 |  |  |  |  |
|  | Control (Contrast) | C C=192, C T=310, T T=107 |  |  |  |  |  |  |  |  |  |  |  |  |
|  | EO versus LO |  | 8.665 | 3 | **0.013*** | 0.195 | 4.293 | 2 | **0.038*** | 0.570 | 0.272 | 4 | 0.762 | 0.953 |
| rs6296 | Overall OCD | G G=119, C G=356, C C=155 | 1.474 | 3 | 0.479 | 0.958 | 0.033 | 2 | 0.856 | 1.000 |  |  |  |  |
|  | Early-onset | G G=47, C G=143, C C=67 | 0.447 | 3 | 0.800 | 0.857 | 0.271 | 2 | 0.603 | 1.000 |  |  |  |  |
|  | Late-onset | G G=70, C G=203, C C=80 | 1.998 | 3 | 0.368 | 1.000 | 0.135 | 2 | 0.713 | 1.000 |  |  |  |  |
|  | Control (Contrast) | G G=132, C G=325, C C=153 |  |  |  |  |  |  |  |  |  |  |  |  |
|  | EO versus LO |  | 0.870 | 3 | 0.647 | 1.000 | 0.764 | 2 | 0.382 | 0.819 | 1.435 | 4 | 0.239 | 0.896 |
| rs13212041 | Overall OCD | T T=350, C T=248, C C=30 | 3.660 | 3 | 0.160 | 1.000 | 0.112 | 2 | 0.738 | 1.000 |  |  |  |  |
|  | Early-onset | T T=141, C T=104, C C=11 | 3.421 | 3 | 0.181 | 0.905 | 0.047 | 2 | 0.828 | 1.000 |  |  |  |  |
|  | Late-onset | T T=192, C T=140, C C=19 | 2.404 | 3 | 0.301 | 1.000 | 0.433 | 2 | 0.511 | 1.000 |  |  |  |  |
|  | Control (Contrast) | T T=350, C T=218, C C=40 |  |  |  |  |  |  |  |  |  |  |  |  |
|  | EO versus LO |  | 0.404 | 3 | 0.817 | 1.000 | 0.026 | 2 | 0.872 | 0.872 | 1.344 | 4 | 0.262 | 0.786 |
| rs4421293 | Overall OCD | G G=574, A G=58, A A=2 | 0.283 | 3 | 0.868 | 0.930 | 0.216 | 2 | 0.642 | 1.000 |  |  |  |  |
|  | Early-onset | G G=233, A G=23, A A=2 | 1.528 | 3 | 0.466 | 1.000 | 0.094 | 2 | 0.759 | 1.000 |  |  |  |  |
|  | Late-onset | G G=323, A G=32, A A=0 | 0.226 | 3 | 0.893 | 1.000 | 0.322 | 2 | 0.571 | 1.000 |  |  |  |  |
|  | Control (Contrast) | G G=547, A G=61, A A=2 |  |  |  |  |  |  |  |  |  |  |  |  |
|  | EO versus LO |  | 0.006 | 3 | 0.997 | 0.997 | 0.083 | 2 | 0.774 | 1.000 | 2.036 | 4 | 0.131 | 0.655 |
| rs17641078 | Overall OCD | G G=476, G C=149, C C=10 | 0.027 | 3 | 0.986 | 0.986 | 0.027 | 2 | 0.870 | 1.000 |  |  |  |  |
|  | Early-onset | G G=193, G C=62, C C=4 | 0.139 | 3 | 0.933 | 0.933 | 0.903 | 2 | 0.342 | 1.000 |  |  |  |  |
|  | Late-onset | G G=268, G C=81, C C=6 | 0.127 | 3 | 0.938 | 0.938 | 0.132 | 2 | 0.716 | 0.976 |  |  |  |  |
|  | Control (Contrast) | G G=459, G C=142, C C=8 |  |  |  |  |  |  |  |  |  |  |  |  |
|  | EO versus LO |  | 0.269 | 3 | 0.874 | 1.000 | 1.477 | 2 | 0.224 | 0.840 | 0.422 | 4 | 0.656 | 1.000 |
| rs3824419 | Overall OCD | C C=157, G C=339, G G=139 | 4.440 | 3 | 0.109 | 1.000 | 3.166 | 2 | 0.075 | 1.000 |  |  |  |  |
|  | Early-onset | C C=57, G C=132, G G=69 | 9.003 | 3 | **0.011*** | 0.165 | 7.153 | 2 | **0.007**** | **0.105** |  |  |  |  |
|  | Late-onset | C C=92, G C=196, G G=68 | 2.941 | 3 | 0.230 | 1.000 | 1.199 | 2 | 0.273 | 1.000 |  |  |  |  |
|  | Control (Contrast) | C C=182, G C=300, G G=129 |  |  |  |  |  |  |  |  |  |  |  |  |
|  | EO versus LO |  | 5.937 | 3 | **0.051** | 0.383 | 2.219 | 2 | 0.136 | 0.680 | 0.644 | 4 | 0.526 | 0.986 |
| rs1062613 | Overall OCD | C C=541, C T=85, T T=8 | 1.565 | 3 | 0.457 | 1.000 | 0.001 | 2 | 0.970 | 0.970 |  |  |  |  |
|  | Early-onset | C C=221, C T=33, T T=3 | 0.854 | 3 | 0.652 | 1.000 | 0.000 | 2 | 0.988 | 1.000 |  |  |  |  |
|  | Late-onset | C C=302, C T=49, T T=5 | 1.430 | 3 | 0.489 | 1.000 | 0.000 | 2 | 0.987 | 1.000 |  |  |  |  |
|  | Control (Contrast) | C C=518, C T=87, T T=4 |  |  |  |  |  |  |  |  |  |  |  |  |
|  | EO versus LO |  | 0.020 | 3 | 0.990 | 1.000 | 0.286 | 2 | 0.693 | 1.000 | 0.310 | 4 | 0.733 | 1.000 |
| rs1176744 | Overall OCD | T T=453, G T=167, G G=14 | 1.015 | 3 | 0.602 | 0.963 | 0.037 | 2 | 0.847 | 1.000 |  |  |  |  |
|  | Early-onset | T T=189, G T=65, G G=5 | 0.778 | 3 | 0.678 | 1.000 | 1.057 | 2 | 0.304 | 1.000 |  |  |  |  |
|  | Late-onset | T T=248, G T=99, G G=7 | 0.211 | 3 | 0.900 | 0.964 | 0.076 | 2 | 0.783 | 0.979 |  |  |  |  |
|  | Control (Contrast) | T T=430, G T=171, G G=9 |  |  |  |  |  |  |  |  |  |  |  |  |
|  | EO versus LO |  | 0.550 | 3 | 0.760 | 1.000 | 1.353 | 2 | 0.245 | 0.735 | 0.129 | 4 | 0.879 | 0.942 |
| rs6265 | Overall OCD | A A=150, G A=339, G G=145 | 0.195 | 3 | 0.863 | 0.996 | 0.008 | 2 | 0.931 | 0.998 |  |  |  |  |
|  | Early-onset | A A=61, G A=131, G G=67 | 0.528 | 3 | 0.768 | 1.000 | 0.018 | 2 | 0.892 | 1.000 |  |  |  |  |
|  | Late-onset | A A=85, G A=198, G G=71 | 1.921 | 3 | 0.383 | 0.958 | 0.140 | 2 | 0.708 | 1.000 |  |  |  |  |
|  | Control (Contrast) | A A=142, G A=320, G G=146 |  |  |  |  |  |  |  |  |  |  |  |  |
|  | EO versus LO |  | 3.261 | 3 | 0.196 | 0.588 | 0.097 | 2 | 0.755 | 1.000 | 0.932 | 4 | 0.395 | 0.988 |
| rs9531519 | Overall OCD | C C=275, T C=291, T T=61 | 1.900 | 3 | 0.387 | 1.000 | 0.272 | 2 | 0.602 | 1.000 |  |  |  |  |
|  | Early-onset | C C=120, T C=111, T T=26 | 0.993 | 3 | 0.609 | 1.000 | 0.379 | 2 | 0.538 | 1.000 |  |  |  |  |
|  | Late-onset | C C=140, T C=176, T T=33 | 4.540 | 3 | 0.103 | 0.773 | 0.002 | 2 | 0.968 | 1.000 |  |  |  |  |
|  | Control (Contrast) | C C=274, T C=263, T T=71 |  |  |  |  |  |  |  |  |  |  |  |  |
|  | EO versus LO |  | 4.234 | 3 | 0.120 | 0.450 | 0.392 | 2 | 0.531 | 0.996 | 0.138 | 4 | 0.871 | 1.000 |
| rs9582391 | Overall OCD | C C=369, C A=225, A A=38 | 1.570 | 3 | 0.456 | 1.000 | 0.337 | 2 | 0.562 | 1.000 |  |  |  |  |
|  | Early-onset | C C=138, C A=103, A A=18 | 0.875 | 3 | 0.646 | 1.000 | 0.776 | 2 | 0.378 | 1.000 |  |  |  |  |
|  | Late-onset | C C=222, C A=111, A A=19 | 5.311 | 3 | 0.070 | 1.000 | 2.223 | 2 | 0.136 | 1.000 |  |  |  |  |
|  | Control (Contrast) | C C=336, C A=240, A A=30 |  |  |  |  |  |  |  |  |  |  |  |  |
|  | EO versus LO |  | 5.759 | 3 | 0.056 | 0.280 | 4.007 | 2 | **0.045*** | 0.338 | 0.086 | 4 | 0.917 | 0.917 |
| rs78312845 | Overall OCD | A A=433, A G=190, G G=10 | 1.070 | 3 | 0.586 | 1.000 | 0.341 | 2 | 0.559 | 1.000 |  |  |  |  |
|  | Early-onset | A A=181, A G=71, G G=5 | 2.553 | 3 | 0.279 | 1.000 | 0.101 | 2 | 0.750 | 1.000 |  |  |  |  |
|  | Late-onset | A A=236, A G=114, G G=5 | 1.098 | 3 | 0.577 | 0.866 | 1.095 | 2 | 0.295 | 1.000 |  |  |  |  |
|  | Control (Contrast) | A A=423, A G=178, G G=7 |  |  |  |  |  |  |  |  |  |  |  |  |
|  | EO versus LO |  | 2.445 | 3 | 0.295 | 0.738 | 0.931 | 2 | 0.335 | 0.838 | 0.863 | 4 | 0.422 | 0.904 |
